# Five recommendations for using large-scale publicly available data to advance health among American Indian peoples: the Adolescent Brain and Cognitive Development (ABCD) Study^SM^ as an illustrative case

**DOI:** 10.1038/s41386-022-01498-9

**Published:** 2022-11-16

**Authors:** Evan J. White, Mara J. Demuth, Andrea Wiglesworth, Ashleigh D. Coser, Brady A. Garrett, Terrence K. Kominsky, Valarie Jernigan, Wesley K. Thompson, Martin Paulus, Robin Aupperle

**Affiliations:** 1 Laureate Institute for Brain Research, Tulsa, OK USA; 2 Oxley School of Community Medicine, University of Tulsa, Tulsa, OK USA; 3 Department of Psychology, University of Minnesota, Minneapolis, MN USA; 4 Cherokee Nation, Tahlequah, OK USA; 5 Center for Indigenous Health Research and Policy, Oklahoma State Universit y Center for Health Sciences, Tulsa, OK USA

**Keywords:** Outcomes research, Social neuroscience, Preclinical research

## Abstract

American Indian and Alaska Native (AIAN) populations have suffered a history of exploitation and abuse within the context of mental health research and related fields. This history is rooted in assimilation policies, historical trauma, and cultural loss, and is promulgated through discrimination and disregard for traditional culture and community knowledge. In recognition of this history, it is imperative for researchers to utilize culturally sensitive approaches that consider the context of tribal communities to better address mental health issues for AIAN individuals. The public availability of data from large-scale studies creates both opportunities and challenges when studying mental health within AIAN populations. This manuscript has two goals; first, showcase an example of problematic use of Adolescent Brain Cognitive Development (ABCD) Study^SM^ data to promulgate stereotypes about AIAN individuals and, second, in partnership with collaborators from Cherokee Nation, we provide five recommendations for utilizing data from publicly available datasets to advance health research in AIAN populations. Specifically, we argue for the consideration of (1) the heterogeneity of the communities represented, (2) the importance of focusing on AIAN health and well-being, (3) engagement of relevant communities and AIAN community leaders, (4) consideration of historical and ongoing injustices, and (5) engagement with AIAN regulatory agencies or review boards. These recommendations are founded on principles from broader indigenous research efforts emphasizing community-engaged research and principles of Indigenous Data Sovereignty and Governance.

## Introduction

Health and mental health research conducted among American Indian and Alaska Native (AIAN)^1^ populations has a history of exploitation and abuse [1–6]. Specifically, AIAN communities have suffered medical abuses, including deliberate spread of smallpox through infected blankets, removal of eyelids (i.e., tarsectomy) to treat trachoma, and illegal sterilization of women and girls without knowledge or consent [5]. AIAN communities have also suffered numerous research abuses including administration of dangerous doses of radioactive iodine without consent, conduct of alcohol use research without consent or appropriate consideration of context, genetic research without informed consent, and unethical handling of blood samples [5]. These grave violations have led to a mistrust of healthcare and research systems by AIAN people. The preferences and priorities of AIAN communities have rarely been incorporated into study development, design, or to guide specific research questions. Few researchers engage with AIAN community partners to provide research oversight or inform the potential benefit and costs to their communities [7, 8]. Although the intent of any given research endeavor may be well-meaning, this does not mitigate the potential for substantial negative consequences [9]. For AIAN communities, risks for research harms extend beyond individuals and include risk to communities as a whole [8, 10].

Potential research harms to AIAN communities are both tangible (e.g., discrimination, stigmatization, impeding social opportunity) and dignitary (e.g., violating collective community rights, disrespecting community values) and require thoughtful consideration of researchers through community engagement [10]. By not engaging and partnering with AIAN communities, researchers risk inadvertently reinforcing negative stereotypes, promoting dubious or discriminatory practices, and supporting nefarious agendas. AIAN communities are best positioned to identify potential consequences and use their knowledge to appropriately influence research decisions. Thus, involving community partners from the beginning of any AIAN research study is essential to ensure culturally appropriate research methods are selected.

As AIAN communities experience a disproportionate burden of mental health concerns, research has focused primarily on understanding symptom development, maintenance, and treatment. However, certain risk factors for poor mental health, (e.g., historical trauma, discrimination) are of particular relevance for AIAN communities and underscore the need for tailored research methodologies placed in appropriate theoretical and empirically-validated frameworks [11–15]. Implementing a culturally sensitive approach to research is perhaps more difficult for large, multi-site, ethnically diverse studies with open access datasets. In these cases, researchers who design the study may not be familiar with, or primarily focused on, which assessments to include to index culturally relevant risk factors. Further, researchers who design the study are not always the same researchers who access, analyze, and interpret the data.

The increasing number of large-scale studies with publicly available datasets requires that the National Institutes of Health (NIH) emphasize data sharing policies that enforce appropriate and culturally informed research practices, such as the draft policy on responsible management and sharing of AIAN participant data [16]. A prominent example of a large-scale dataset currently publicly available for researchers to analyze is the Adolescent Brain Cognitive Development (ABCD) Study^SM^, launched by the National Institute on Drug Abuse (NIDA) in partnership with other intramural and extramural research programs [17]. The ABCD Study^®^ is the largest study of human brain development in the United States, aiming to understand neurocognitive development and associated factors with a focus on development of substance misuse and related conditions. The study includes multi-method (e.g., self-report, clinical rating, bioassay, neuroimaging) longitudinal data to track biological and behavioral development through adolescence into early adulthood [18]. Given the size of the sample (*n* = 11,880 at baseline) and intent to reflect the sociodemographic characteristics of the USA [19], AIAN youth and families are represented in this study on an unprecedented scale for developmental research.

With thoughtful community collaboration, the ABCD Study^®^ data could serve as a substantial resource for AIAN communities to leverage in efforts to promote mental health and wellness of their youth. However, substantial AIAN participation in the ABCD Study^®^ also raises the possibility for researchers to conduct inappropriate research with adverse effects for AIAN communities. If problematic research practices are employed, these data represent a large source of threat to AIAN communities. Thus, it is imperative that researchers using ABCD data (or other publicly available datasets) to examine questions relevant to AIAN communities conduct work that ensures (1) high-level scientific rigor expected when working with large-scale studies [20] and (2) benefit and not harm to AIAN communities [8]. Moreover, researchers accessing publicly available datasets (e.g., ABCD Study^®^) have a responsibility to acknowledge and critique improperly conducted research to mitigate irresponsible and harmful research practices. This responsibility also is carried by independent research reviewers, editors, and journal publishers who evaluate and disseminate such works [21].

The goals of the current paper are two-fold. First, we provide a critical response to Assari [22], as an example of how availability of large-scale data such as that from the ABCD Study^®^ can be used improperly in such a way as to potentially do harm. Second, in partnership with expert behavioral health researchers from the Cherokee Nation (CN), we offer recommendations for engaging in research with the data from large-scale publicly available data including the ABCD Study^®^. The Cherokee Nation provides IRB oversight for the ABCD Study^®^ site at the Laureate Institute for Brain Research (LIBR) in Tulsa, Oklahoma and representatives from CN sit on the community advisory board for the LIBR site. This partnership imparts a unique responsibility and opportunity to provide the commentary and recommendations contained herein. Although the recommendations are focused on the ABCD Study^®^, they are also relevant for other emerging population-based datasets available (or soon to be available) for analysis such as “All of Us” Study [23] and the HEALthy Brain Child Development (HBCD) Study [24].

## Critique of Assari [22]

Recently Assari [22], published what we strongly consider to be an inappropriate interpretation of the ABCD Study^®^ data, suggesting key protective factors against high body mass index (BMI) in childhood were not protective among AIAN populations. The paper [22] serves as an instructive example of how publicly available datasets pose disproportionately high risks for misinterpretation in AIAN populations. Furthermore, as AIAN researchers and allies interested in promoting research that prioritizes AIAN community well-being, it is our duty to correct inappropriate research practices and contextualize findings that have been presented in an uninformed fashion. Thus, we cover three points salient to a critique of the Assari (2020) paper: (1) substantial literature relevant to BMI in AIAN populations absent in the theoretical rationale; (2) misinterpretation of statistical analyses; and (3) lack of community engagement.

### Theoretical rationale

The first and foremost concern is the dubious validity of the presented rationale. Specifically, the foundational theoretical model – previously developed by the author – suggests that minoritized communities experience reduced health benefits from protective factors. This model was not developed with AIAN communities, nor has the conceptual framework been validated with these populations. Moreover, the study did not take into account the large body of extant research on obesity in AIANs [25]. It has been noted for over a decade that metabolic disease prevalence has been increasing in AIAN populations and that risk factor assessments commonly used are inadequate to capture variance related to increasing rates. Specifically, common assessments suffer from low response rate; no consideration of rural, urban, or reservation based sampling, and limited sociodemographic variability [26]. Notably, the ABCD Study^®^ was designed with specific retention efforts in mind [27] and sampling procedures were aimed at recruiting a sample representative of the general U.S. population [19]. Although the ABCD Study^®^ was not initially designed to address questions relevant only to AIAN populations, the published design elements of the ABCD study facilitate an articulation of how these considerations may impact conclusions drawn from analysis of the data. Such considerations are absent or minimally alluded to in the limitations reported in the Assari paper [22].

Another important construct missing from the presented rationale [22] is food insecurity, which has been identified as a key risk factor for obesity [28] and is a disproportionate burden among AIAN communities [29]. Increased burden of food insecurity likely results from historically discriminatory practices, inadequate availability of culturally appropriate services (e.g., food sovereignty and security interventions) [30, 31], and socioeconomic disadvantage. CN health system partners also report that limited access to fresh produce is a common barrier to healthy eating in some communities. Additionally, obesity rates are higher for AIAN populations who report healthy food prices as a significant barrier to consumption or those who rely on alternative food stores (e.g., convenience stores, gas stations) as a routine source of food [32].

Considering the broad health disparities among AIAN populations, properly conducted research is critical to supporting better health outcomes in these communities. Indeed, many efforts are underway which employ multi-level, community-engaged methodologies to advance health in AIAN populations (i.e., Intervention Research to Improve Native American Health [IRINAH]) [33]. Research seeking to establish or test a theory should include interdisciplinary perspectives that span the areas of research relevant to the theory in question [2] and include substantial community engagement [7]. Although not an exhaustive review of relevant literature, these points are illustrative of an established field of research aimed at delineating nuance related to metabolic health disparities among AIAN populations. This crucial research context is completely neglected in the paper [22], calling into question the validity of the generic theoretical rationale presented and interpretations of the data.

### Inappropriate interpretation of statistical analyses

The work in question also employs statistical analyses of questionable validity, thus precluding the ability to draw meaningful conclusions. Specifically, the analysis sample is extremely imbalanced across comparison groups, the non-Hispanic White (NHW) group having 8517 participants compared to 63 in the AIAN, Native Hawaiian, and Pacific Islander (NHPI) group. It is unclear why the AIAN/NHPI group is so small relative to what would be expected given baseline demographics reported for the ABCD Study^®^ (>280 AIAN depending on categorization of multi-racial individuals) [34] as the reporting of sampling procedures [22] lacks necessary detail to determine how the sample was identified. This raises serious concerns about the robustness of the analytic approach to the influences of unequal variance. Moreover, for the main interaction effects reported (i.e., education and income) the cell size in the AIAN/NHPI group at the highest levels of protective factors was *n* = 4 (post-graduate degree) and *n* = 8 (family income ≥ $100 K) compared to *n* = 3930 and *n* = 4842 in the NHW group, respectively. Mean estimates derived from such small samples are very likely to be unreliable and not representative of the broader AIAN population. Furthermore, reporting such small cell sizes increases the potential risks to participant confidentiality, particularly in populations that are more readily identifiable from study variables [35].

There is substantial cultural, historical, and ethnic heterogeneity within the AIAN/NHPI group that is ignored in the work in question. There are 574 federally recognized AIAN nations in the United States without accounting for heterogeneity among NHPI peoples. Similarities and dis-similarities across these populations will depend on the research question and relevant policies for population(s) of interest. Collapsing AIAN/NHPI participants into one collective group has been referred to as “ethnic gloss”, and creates substantial problems for external validity and replicability of findings [36, 37]. It is unreasonable to expect the relatively small AIAN/NHPI group (*n* = 63) to be representative of this collection of culturally, geographically, politically, and linguistically distinct populations. Additionally, the interpretation of statistical associations as “causal effects” is inappropriate for cross-sectional observational data [20]. The effect sizes reported are less than one-quarter of 1% of variance in BMI. Merely rejecting a null hypothesis for a correlation is insufficient grounds for making broad generalizations about mechanisms or for testing unsupported, nonspecific causal theories [20]. In short, the purported conclusions of the work in question [22] are unwarranted for three critical reasons: 1) small sample size with dubious generalizability; 2) lack of culturally appropriate measures to differentiate specific hypotheses regarding the link between BMI and parental education/household income and 3) lack of alternative explanatory models for the observed associations in cross-sectional observational data.

### Lack of community engagement

Recommendations for conducting research with AIANs call for substantial involvement of community members in the research process due to rich diversity of AIAN populations as well as prior research endeavors that have harmed these communities [7, 38–41]. Such engagement ensures that research questions, variables of interest, measurement strategies, and interpretations make sense within the population of interest; lack thereof increases the potential for erroneous conclusions. Community based participatory research (CBPR) is the gold standard for conducting health disparities research in general and specifically in AIAN communities [7]. Prior research has provided recommendations to contextualize CBPR principles within AIAN communities [7] and showcased examples of research partnerships employing these approaches [38]. Recent work has also highlighted foundational principles for the ethical conduct of research in tribal communities [8, 9]. Specifically, two guiding principles are identified (1) sovereignty, to ensure both self-determination and oversight in the research process including regulatory review and (2) solidarity, to align the research with the well-being of the community [8]. The work in question [22] did not report community engagement or regulatory review, thus increasing the likelihood of erroneous conclusions. The lack of engagement also precluded any potential benefit to the research from AIAN community knowledge did not enable consideration of how the communities may benefit or be harmed by the work conducted. Finally, the unfounded conclusions presented [22] risk extreme harm as they could be cited as a rationale for policy decisions which reduce availability of resources that support health, education, and employment opportunities in AIAN communities.

### Summary of critique

Research conducted to address health disparities in AIAN communities – including those concerning metabolic diseases – are laudable. However, such work should be appropriately culturally sensitive and consider the perspectives of the communities involved to increase the validity of the findings. The potential for promulgating harmful stereotypes and public policy decisions based on dubious research underscores the general imperative of rigorous research practices in large-scale data projects. Best practice in research among AIAN communities calls for significant community involvement (e.g., CBPR) even in the case of otherwise scientifically and quantitatively rigorous methodologies. The paper in question [22] suffers from three critical flaws (1) lack of appropriate context from extant literature regarding specific risk factors relevant to AIAN populations, (2) flawed interpretation of statistical analyses, and (3) lack of engagement with community members and inclusion of community knowledge to inform interpretations.

## Recommendations for analyzing publicly available AIAN participant data

Recommendations from CBPR include engaging with AIAN community members in the generation of research questions at the early stage of study design [7, 9]. This enables community partners to help drive the research process from the outset. Due to the scope and ongoing nature of many large-scale publicly available datasets including ABCD, such a degree of community involvement is not possible for all researchers who may engage in analysis of the resultant data. However, this does not alleviate the responsibility for researchers examining AIAN participant data from publicly available data to employ an informed and respectful approach to the work. For example, the LIBR site of the ABCD study^®^ engaged with CN-IRB to provide oversight on recruitment and study protocols when initiating the study. Representatives from CN sit on the advisory board for the study and CN-IRB provide ongoing oversight of the protocol, modifications, and dissemination (i.e., manuscripts and presentations) from LIBR researchers. In collaboration with CN partners, we provide a set of brief recommendations for conducting analysis on large-scale, publicly available datasets (e.g., ABCD, All of Us, HBCD) to facilitate research that prioritizes well-being of AIAN communities. Additionally, these recommendations are informed by on-going work of researchers conducting community engaged research with AIAN communities in many different settings [42, 43]. Importantly, the *generators* (i.e., funding agencies, researchers designing protocols, and individuals collecting the data) and *users* (i.e., researchers analyzing and publishing the data) of large-scale publicly available AIAN participant data carry a responsibility to apply the following recommendations to their work.

1. *Consider heterogeneity of large-scale AIAN samples:* A key consideration in examining AIAN samples from large-scale publicly available data is the heterogeneity across these communities. The AIAN population comprises 574 federally recognized sovereign nations in the United States, each representing distinct cultural groups with unique and overlapping history, language, traditions, spiritual practices, and foods. This complexity is compounded by modern distributions of these populations across rural and urban settings as well as living on or off reservations. Understanding the potential influence of such heterogeneity on research findings is important to avoid overgeneralization and ethnic gloss [37]. With respect to publicly available datasets, *users* should carefully consider in their analysis plans and transparently discuss within their publications the impact of such heterogeneity on their research questions. Notably, many large-scale studies do not collect tribal affiliation information to respect tribal autonomy as collecting tribal affiliation requires additional responsibilities (e.g., permission from named tribes). The strength of large-scale data can be leveraged only insofar as researchers consider the degree to which variables of interest are shared (e.g., historical trauma, sociopolitical risks associated with colonization) or distinct (e.g., cultural, socioeconomic, geographic, linguistic factors) across these heterogeneous communities. Thus, *generators* of these datasets can ensure adequate data collection to characterize the potential heterogeneity with respect to such variables without the need to collect tribal affiliation.
2. *Prioritize advancement of health and wellbeing in AIAN communities:* The foundational motivation for research should be a beneficial impact on communities the research is meant to serve. Often the nature of science is aimed at potential long-term benefits through incremental research endeavors. Although this is laudable and a necessary feature of much scientific work, research can be designed and executed with an emphasis on immediate direct and indirect benefits to communities. Many AIAN communities that partner in research are overburdened and under resourced with respect to mental health care and related services. Carelessly designed research may exacerbate such difficulties [9]. In concert with study goals, protocols should include design elements that increase resources (e.g., protected time, funding, personnel) and bolster sustainability for tribal research collaborators. Additionally large-scale studies should include efforts to increase capacity for AIAN communities to engage with publicly available data generated by these protocols. For large-scale study *generators*, this could include dedicated funding opportunities, trainings, and data analytic infrastructure made available to AIAN tribal entities. Moreover, *users* analyzing extant publicly available data also should prioritize advancement of health and well-being of AIAN communities. Specifically, it is important that *users* employing publicly available AIAN participant data consider contextual factors, such as community resource burden, how to provide benefit to community partners (e.g., funding, personnel time, training, communicating findings of research) through the research process surrounding their research questions. Furthermore, *users* should conduct analyses with an understanding of how analyses may advance or hinder public policy and public perceptions relevant for AIAN communities. This concern is underscored by the potential policy impact of large-scale open access datasets [44] and neuroscience research more broadly [45]. Prior research has demonstrated health disparities in AIAN communities are specifically linked to a history of problematic public policy including consistent underfunding of Indian Health Service(IHS) [46–50]. Thus, inappropriate research using large-scale open access data may exacerbate resource inequities resultant from public policy decisions. Moreover, it has been repeatedly demonstrated that inappropriate research promulgates negative stereotypes [43, 45, 51, 52] that are associated with harm psychological consequences for AIAN people [53, 54].
3. *Facilitate community engagement at each stage of the research process:* CBPR [38] approaches represent the gold standard research method in AIAN communities. Researchers examining publicly available datasets should engage with community partners (e.g., local tribal communities) early in the research development process. This collaboration ensures the research is of interest to and serves a need for the community, especially if the research question is identified by community partners rather than academic collaborators. Community partners should also be involved in interpretation of findings to promote an emic approach to understanding research results and an avenue to incorporate deep community knowledge and Indigenous ways of knowing into the study findings. It also is important to disseminate the results beyond scientific outlets, by communicating results in an understandable fashion to community members and institutions. This dissemination should prioritize bidirectional information sharing; thus, promoting understanding of findings and facilitating feedback from the community partners. This circular information flow enhances the impact of research findings, reinforces community engagement in research efforts, and improves sustainability of research partnerships that benefit communities.
  The scope and scale of national, publicly available studies may complicate and even preclude deep engagement with all potential AIAN communities of interest. However, this does not absolve *generators* and *users* of the responsibility to make good faith efforts to establish appropriate research protocols including design, recruitment, as well as data collection, storage and sharing agreements. Such efforts of the *generators* may include (1) requests for information (RFI) from funding agencies sent directly to tribal institutions, (2) public comment periods, and (3) convening national work groups of AIAN community representatives and their research collaborators. Results of such approaches can then be made publicly available in study descriptions. *Generators* can also consider creating a protected access repository of relevant research findings, requiring *users* to submit lay summaries of their findings in addition to preprints and relevant analytic scripts or methods associated with their work that AIAN communities and agencies would have access to. Furthermore, *generators* could require that data use agreement applications from *users* specify plans for community engagement and/or include a letter of support from community partners to access publicly available AIAN participant data. Thus, *users* should engage with community partners to collaborate on projects that involve analysis of AIAN participant data from largescale publicly available data at the earliest stage of the process that the *user* is involved in and find avenues for dissemination of the research back to the community. As a first step in this direction, researchers should seek out collaborators who have expertise in community engaged research with AIAN populations.
4. *Consider the impact of social injustices on study variables:* Colonization has led to widespread historical trauma and loss for AIAN people [55–57]. Moreover, AIAN communities are negatively impacted by intergenerational transmission of trauma [58] and discrimination [57, 59, 60]. The pernicious impact of these factors on mental health disparities is widespread and multifaceted. Although this precludes specific prescriptive recommendations, *users* should examine the influence of such variables on findings of interest. If unable to model such influences, it is incumbent on *users* to contextualize findings with this limitation. This is especially necessary in the examination of biological data within any particular racial group, as race is not a biological variable [61], nor is it appropriately conceptualized as a causal variable [62]. This is of particular sensitivity in AIAN populations due to the history of problematic uses of such data [5]. Absence of such considerations leads to erroneous conclusions and promulgates uninformed harmful stereotypes of AIAN people. This concern is enhanced in large-scale publicly available datasets considering the high-level policy implications of such work for health disparities research. Moreover, applying the Belmont Report’s [63] principle of justice to health disparities research, *generators* of population level studies should employ sampling practices that reflect relative disease burden across demographic categories rather than census level population data. *Generators* of such studies should engage directly with AIAN communities to ensure the study addresses meaningful questions for these populations, assesses all relevant constructs, and uses appropriate measurements.
5. *Engage with tribal research regulatory infrastructure:* Although important, community engagement is insufficient to ensure ethical research practices [8]. Research regulation within AIAN communities by tribal governments is rooted in tribal sovereignty;[64] thus, researchers should comply with regulatory infrastructure of their community collaborators (e.g., IRB, tribal council, memorandums of agreement). *Users* should engage with tribal regulatory bodies for consultation and or oversight of analysis focused on AIAN participant data from large publicly available datasets. Prior work has put forth a framework and principles of ethical conduct of research with Indigenous communities [65] which may serve as a guide for researchers when examining publicly available AIAN data. The design of future large-scale studies which include AIAN samples, would benefit from coordinated efforts of *generators (e.g., funding agencies)* to establish regulatory bodies comprising tribal representatives and experts in community engaged research in AIAN populations. These entities may be tasked with the oversight of design, implementation, data storage, and data sharing agreements of publicly available data. Such efforts should be informed by recommendations for Indigenous Data Governance (IDG) and Indigenous Data Sovereignty (IDS) [66] consistent with the United Nations Declaration on the Rights of Indigenous Peoples (UNDRIP) [67]. Consistent with the recommendation regarding community engagement, ongoing studies with publicly available data could adjust data use certifications to include requirements consistent with recommendations for operationalizing IDS and IDG [68, 69].
  In addition to the recommendations for researchers, self-determination of AIAN communities within publicly accessible datasets would be greatly enhanced by community lead research calls published by tribes themselves. Specifically, tribal entities interested in leveraging publicly available data could solicit proposals from potential collaborators and organizations with research infrastructures that may enhance the ability of communities to conduct such work. Lastly, funding agencies could partner with tribes to produce funding announcements commensurate with research priorities derived from the community directly.

## Conclusion

Research conducted among AIAN populations has often resulted in harm or lack of benefit to communities in which the research was conducted. The potential for harm or lack of benefit is amplified in large-scale studies with publicly available data. Recent work is illustrative of this risk [22]. Thus, it is important that culturally appropriate research practices are employed by researchers using these data. These studies provide an opportunity to advance our understanding of the health and well-being of AIAN peoples, but only if conducted with appropriate consideration of cultural context. Specifically, it is crucial for such research to (1) consider heterogeneity of the communities represented, (2) prioritize the promotion of AIAN health and well-being, (3) engage relevant communities and AIAN community members, (4) consider the impact of social injustices, and (5) engage with AIAN research regulatory agencies. Specific recommendations above include considerations for the design and conduct of large-scale studies and for researchers accessing and analyzing publicly available data. The current work is driven by a commitment to the ethical principal of solidarity [8] in research; specifically, in the relationship between the LIBR ABCD site and CN. Although the recommendations presented are specific to AIAN populations and incorporate practical nuance of working with sovereign nations, these recommendations could be adapted for researchers interested in working with other marginalized populations represented in large-scale publicly available datasets.

## Footnotes

1 It is important to note that terms such as American Indian/Alaska Native (AIAN), Native, Indigenous, First Peoples, have often been used interchangeably and it is most respectful to use terminology individuals and communities prefer. For the current study we use the term AIAN to refer to individuals self-reported to be from heterogenous U.S. tribal nations (e.g., federal, state, or non-recognized) in order to be consistent with language used in the Adolescent Brain and Cognitive Development study and National Institutes of Health research announcements.
